# Salmonella-Related Septic Arthritis in an Immunocompetent Adult: A Case Report

**DOI:** 10.7759/cureus.21030

**Published:** 2022-01-08

**Authors:** Miral H Gharib, Seham Alebbi, Farah Rashid, Maab F Elhaj, Fathima z Zahirb, Samar AL Emadi

**Affiliations:** 1 Rheumatology, Hamad Medical Corporation, Doha, QAT; 2 Internal Medicine, Hamad Medical Corporation, Doha, QAT

**Keywords:** monoarthritis, inflammatory, hip joint, septic arthritis, salmonella

## Abstract

*Salmonella* is a well-known cause of foodborne illness, commonly resulting in gastroenteritis, bacteremia, and focal infections. *Salmonella* septic arthritis is a rare entity with cases mostly prevalent in patients with sickle cell disease, immunocompromised state, and advanced age. Here, we present a case of *Salmonella* septic arthritis in a previously healthy young gentleman with no risk factors who presented with fever, skin rash, abdominal pain, and left hip pain. Initial laboratory findings showed high inflammatory markers and negative blood culture. MRI of the left hip joint showed effusion and features of inflammatory changes. The diagnosis was confirmed by joint aspirate culture growing *Salmonella *B. Furthermore, he underwent joint arthrotomy; pus was drained and treated with an antibiotic. Subsequently, the patient responded to treatment with marked clinical recovery.

## Introduction

It is well known that *Salmonella* is one of the leading causes of foodborne bacterial diseases worldwide. It is transmitted via the fecal-oral route and commonly results in gastroenteritis, bacteremia, and other localized infections [1]. In comparison to other gram-negative bacteria, *Salmonella* is rarely encountered as a cause of osteoarticular infections. There are well-described reports of septic arthritis secondary to *Salmonella* particularly in individuals who are immune-compromised or have a history of sickle cell disease, systemic lupus erythematosus (SLE), or prosthetic joints [2,3,4]. However, *Salmonella* arthritis in a native adult joint of healthy individuals is a rare phenomenon that has not been well established in the literature. Here we describe a case of septic arthritis of the hip joint caused by group B *Salmonella* in an immune-competent individual with no underlying comorbidities. In addition to its rarity, this case is presented to emphasize the unusual manifestations of group B *Salmonella*.

## Case presentation

**Figure 1:**
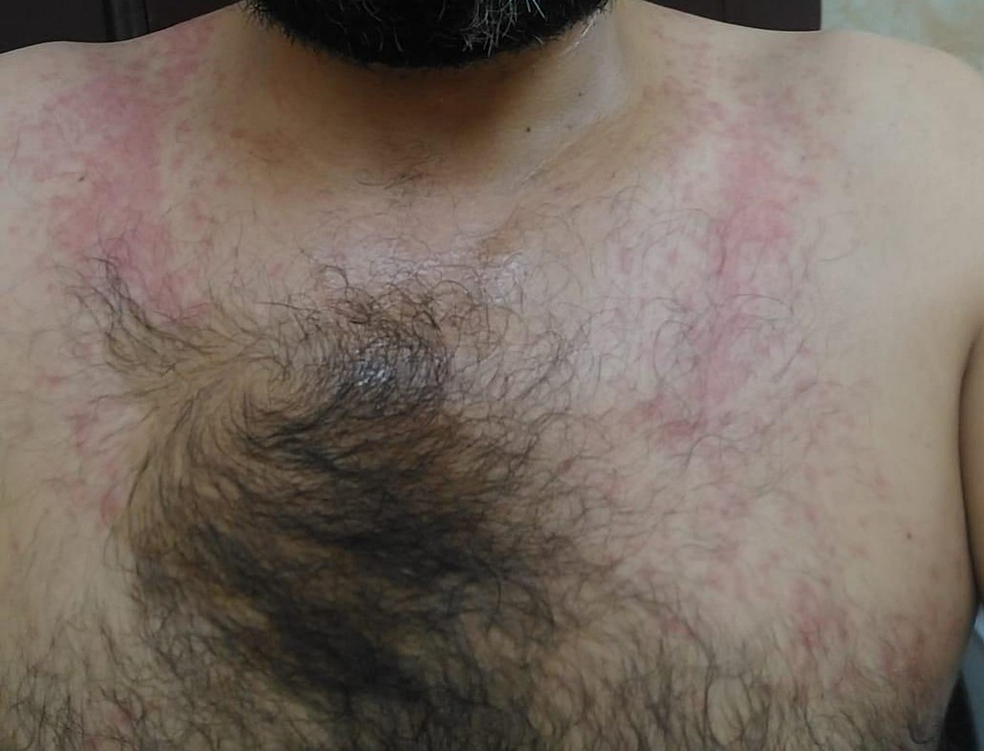
An erythematous rash over the chest.

**Figure 2:**
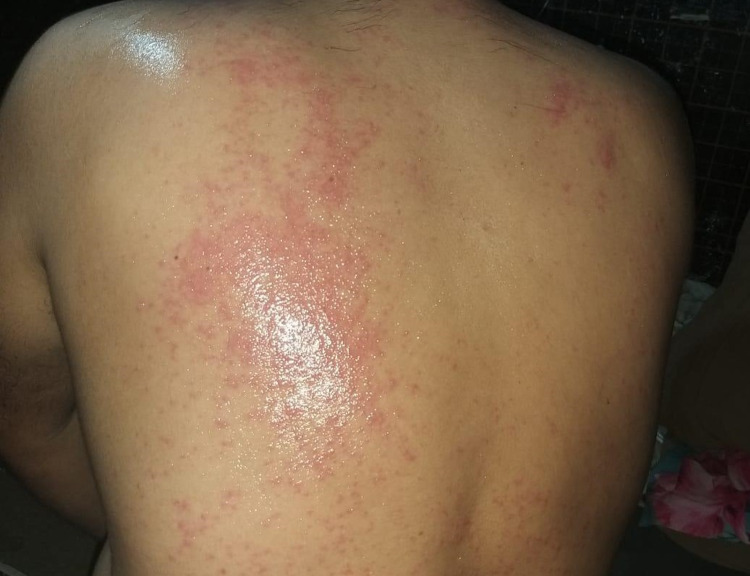
An erythematous rash over the back.

Written informed consent was obtained from the patient for the publication manuscript and images. A 37-year-old Syrian male with no comorbid conditions presented to the emergency department with complaints of lower back pain radiating to the left leg for 10 days. Detailed history taking revealed that for two weeks prior to hospital admission, he developed erythematous skin rash involving the face and trunk, followed by painful oral ulcers. Later on, he started to become febrile and developed left hip pain. Hip pain was dull, aching in quality, radiating to the groin of moderate intensity, and associated with an inability to bear weight, but there was no morning stiffness, no other joint involvement, no history of similar presentation, and no history of trauma or surgery. The patient also denied any history of nausea, vomiting, constipation, or diarrhea. On review of systems, there was no cardiovascular, pulmonary, renal, or other major organ involvement. There was also no relevant past or family medical history. On arrival, his vital signs were as follows: blood pressure of 120/80 mm Hg, temperature (oral) of 38°C, and respiratory rate of 18 breaths per minute. Upon examination, the patient was found to have discrete, painful shallow, circular aphthous oral ulcers associated with blistering rash around the lips. He also had an erythematous maculopapular rash over the chest and back shown below (Figures 1, 2). In addition, there was restricted, painful flexion, internal and external rotation of the left hip, right hip joint, and examination was normal. Rest of the systemic examination was unremarkable. Routine labs were conducted, and initial laboratory data are given in Table 1, which show leukocytosis, mildly elevated liver function tests, and high inflammatory markers. Furthermore, X-ray of his left hip showed no bony pathology or fracture. In view of fever, oral ulcer, herpes labialis, erythematous rash, and hip joint pain, he was started on ceftriaxone for suspected gonorrhea infection. However, patient symptoms did not improve and left hip pain worsened; therefore, the rheumatology team was consulted for possible reactive arthritis for which he was started on NSAIDs (non-steroidal anti-inflammatory drugs).

**Table 1:** Initial laboratory findings CBC, complete blood count; WBC, white blood cells; ALT, alanine transaminase; AST, aspartate transaminase; ALP, alkaline phosphatase; CRP, C-reactive protein; ESR, erythrocyte sedimentation rate

CBC	Result (normal value)
Hemoglobin	14.9 g/dL (13-17g/dL)
WBC	6.6 x10^3^/uL ( 4 x10^3^/uL-10 x10^3^/uL)
Platelet	208 x10^3^/uL (150 x10^3^/uL-400 x10^3^/uL)
Liver function test	
ALT	142 U/L (0-41 U/L)
AST	99 U/L (0-41 U/L)
ALP	154 U/L (40-129 U/L)
Bilirubin	16 umol/L (0-21 umol/L)
Inflammatory markers	
CRP	136.9 mg/L (0-5 mg/L)
ESR	39 mm/hr (2-28 mm/hr)

**Figure 3:**
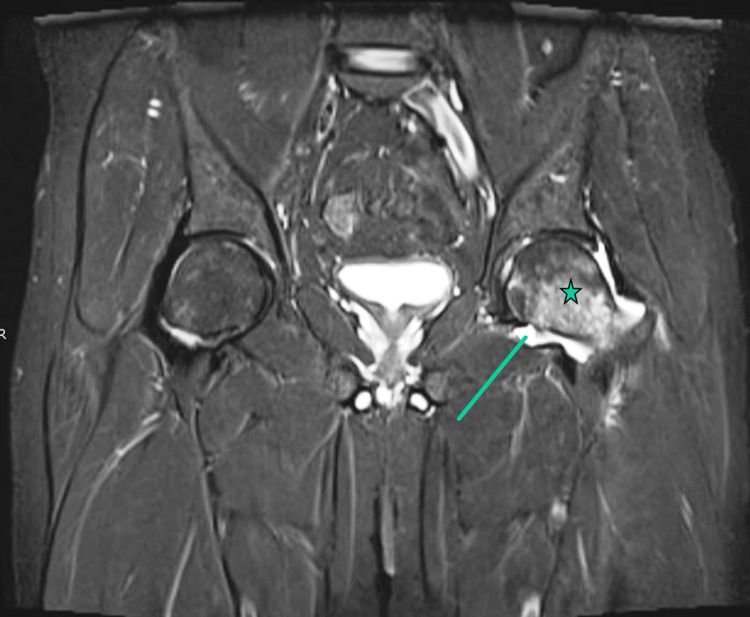
This is a fluid-sensitive image (STIR sequence) showing left hip joint effusion (arrow) and bone marrow edema (asterisk). STIR, short inversion time inversion recovery

Workup for reactive arthritis (hepatitis serology, parvovirus PCR, brucella serology, chlamydia, gonorrhea, cytomegalovirus [CMV], Epstein-Barr virus [EBV], adenovirus, and HIV) was negative, including blood cultures. On follow-up, his oral ulcer, skin rash improved but hip pain persisted. For further evaluation of persistent hip joint pain, MRI of the left hip joint was performed, which revealed joint effusion and left proximal femur bony edema, findings suggestive of infective/inflammatory process (Figure 3).

Left hip joint aspiration was performed by interventional radiology and straw-colored fluid was sent for analysis, cytology, and culture. Fluid analysis revealed turbid fluid with white blood cell (WBC) count of 3,875/mL with 49% neutrophil. Synovial fluid grew gram-negative bacilli (*Salmonella* B). The patient was maintained on ceftriaxone 2 g intravenous (IV) daily. He underwent arthrotomy, 30 mL of inflammatory fluid was drained, the joint was washed out, a drain was inserted, which was removed on the third day, and physiotherapy was initiated. Afterward, he started to improve significantly with restoration of fully functional joint, and inflammatory changes subsided. The patient was discharged with a plan of two to three weeks of ceftriaxone IV as per infectious disease (ID) team recommendation.

## Discussion

*Salmonella* belongs to a class of gram-negative bacteria. Two species of *Salmonella* are present: typhoidal and non-typhoidal. The most common source of infection is ingestion of poultry, beef, and eggs; however, direct or indirect contact with reptiles and ingestion of snake-based products (such as meat) have also been reported [5].

Typhoidal species are notably known for causing enteric fever. Non-typhoidal species infection usually manifests as gastrointestinal ailment [6]. Extraintestinal manifestations of *Salmonella *infection include sepsis and involvement of any major organ systems. *Salmonella* infection is commonly reported in children [7]. Patients with an immunocompromised condition such as sickle cell, malignancy, antimicrobial therapy, use of immunosuppressive agents [2], surgical implants, age more than 80 years [3], SLE [4], human immunodeficiency virus (HIV) [8], and diabetes [9] are predisposing features for systemic involvement.

*Salmonella* infection of the bones and joints is rare, and few cases have been reported [3,4,9-12]. As previously described, *Salmonella* often causes monoarthritis, with the hip joint being predominantly involved [4]. Hematogenous spread is the leading cause of developing *Salmonella* septic arthritis. However, blood cultures are often positive in only 10-15% of cases [11].

The novelty of our case is that our patient developed *Salmonella* septic arthritis without any predisposing factors. He presented with abdominal pain, fever, rash, and back pain. Initial labs showed high inflammatory markers and negative blood culture. In the absence of risk factors and obscure symptoms, septic arthritis was not taken into initial consideration. Afterward, the patient developed severe hip pain and limping, which raised the concern for septic arthritis. MRI showed feature of an inflammatory process. Diagnosis was confirmed by hip arthrotomy, and joint fluid culture grew *Salmonella* B, although synovial fluid analysis revealed turbid fluid with only a low WBC count of 3,875/mL with 49% neutrophil.

Management of *Salmonella* osteoarticular infection is controversial, and the variable duration of treatment is available after at least four to six weeks of susceptible antibiotic and sometimes demands surgical debridement [12,13]. Commencement of early treatment in septic arthritis is crucial to prevent sequelae and to warrant early restoration of a fully functioning joint. Our patient underwent left hip arthrotomy and pus was drained, and he was started on ceftriaxone IV. Subsequently, the patient was able to ambulate and showed marked improvement. This case highlights the importance of screening for typhoidal infection in patients who present with fever, monoarthritis in the presence of abdominal pain, and skin rash.

## Conclusions

In conclusion, *Salmonella* septic arthritis of the hip is a rare presentation, especially in non-debilitated adults. Thus, physicians need to be aware of this unusual presentation in young healthy patients. Moreover, as in our patient, the joint motion restriction and typical inflammatory signs seen in early septic arthritis can be delayed in *Salmonella*-related infections. Therefore, each patient with clinical suspicion of septic arthritis must be thoroughly investigated in a timely manner, and antibiotic therapy along with surgical decompression should be performed at the time of diagnosis in order to prevent permanent joint dysfunction.

## References

[1] Boyle EC, Bishop JL, Grassl GA, Finlay BB (2007). Salmonella: from pathogenesis to therapeutics. J Bacteriol.

[2] Hohmann EL (2001). Nontyphoidal salmonellosis. Clin Infect Dis.

[3] Lo I, Chang HC (2018). Salmonella septic arthritis in a patient with a hip implant: a case report. Int J Gerontol.

[4] Huang JL, Hung JJ, Wu KC, Lee WI, Chan CK, Ou LS (2006). Septic arthritis in patients with systemic lupus erythematosus: salmonella and nonsalmonella infections compared. Semin Arthritis Rheum.

[5] Ispahani P, Slack RC (2000). Enteric fever and other extraintestinal salmonellosis in University Hospital, Nottingham, UK, between 1980 and 1997. Eur J Clin Microbiol Infect Dis.

[6] Linam WM, Gerber MA (2007). Changing epidemiology and prevention of Salmonella infections. Pediatr Infect Dis J.

[7] Zaidi E, Bachur R, Harper M (1999). Non-typhi Salmonella bacteremia in children. Pediatr Infect Dis J.

[8] Fernández Guerrero ML, Ramos JM, Núñez A, Cuenca M, de Górgolas M (1997). Focal infections due to non-typhi Salmonella in patients with AIDS: report of 10 cases and review. Clin Infect Dis.

[9] Gondusky JS, Gondusky CJ, Helmers SW (2009). Salmonella osteomyelitis in new-onset diabetes mellitus. Orthopedics.

[10] Van Cappelle, H. G., Dick Veenendaal, and Pieter L. de Vogel (1995). Salmonella panama osteomyelitis in an otherwise healthy patient. A case report.. Clin Orthop Relat Res.

[11] Al Nafeesah AS (2015). Nontyphoidal Salmonella septic arthritis of the elbow in a healthy infant. Pan Afr Med J.

[12] Schneider L, Ehlinger M, Stanchina C, Giacomelli MC, Gicquel P, Karger C, Clavert JM (2009). Salmonella enterica subsp. arizonae bone and joints sepsis. A case report and literature review. Orthop Traumatol Surg Res.

[13] Uygur E, Reddy K, Ozkan FÜ, Söylemez S, Aydin O, Senol S (2013). Salmonella enteridis septic arthritis: a report of two cases. Case Rep Infect Dis.

